# Dissolving Microneedle Patches for Transdermal Insulin Delivery in Diabetic Mice: Potential for Clinical Applications

**DOI:** 10.3390/ma11091625

**Published:** 2018-09-05

**Authors:** Chih-Hao Chen, Victor Bong-Hang Shyu, Chien-Tzung Chen

**Affiliations:** 1 Department of Chemical and Materials Engineering, Chang Gung University, Taoyuan 333, Taiwan; chchen5027@gmail.com; 2 Craniofacial Research Center, Department of Plastic and Reconstructive Surgery, Chang Gung Memorial Hospital at Linkou, Chang Gung University, College of Medicine, Taoyuan 333, Taiwan; vbshyu@yahoo.com.tw; 3 Department of Plastic and Reconstructive Surgery, Chang Gung Memorial Hospital at Keelung, Chang Gung University, College of Medicine, Keelung 204, Taiwan

**Keywords:** microneedle, polymer, transdermal, insulin, diabetes mellitus

## Abstract

In this study, dissolving polymeric microneedle (MN) patches composed of gelatin and sodium carboxymethyl cellulose (CMC) were used to localize insulin. Their in vitro skin insertion capabilities were determined using tissue-marking dye to stain the skin after patches removal. Scanning electron microscopy (SEM) was used to determine changes in the MNs over time, and optical coherence tomography (OCT) was used to monitor their real-time penetration depth. Confocal microscopy images revealed that rhodamine 6G gradually diffuses from the puncture sites to deeper dermal tissue. Using an in vivo imaging system (IVIS), skin areas that received FITC-insulin-loaded MNs were found to present strong fluorescent signals that greatly decreased 1 h after application. Results show that dissolving MNs rapidly release FITC-insulin, and it then gradually diffuses into the skin. This study verifies that using a gelatin/CMC MN patch for insulin delivery achieves satisfactory relative bioavailability compared to a traditional hypodermic injection and can be a promising delivery device for poorly permeable protein drugs such as those used to treat diabetes. Insertion tests on human cadaveric skin demonstrate that dissolving MNs could serve as efficient devices for transdermal drug delivery in clinical practice and that the volar aspect of forearm skin is the ideal location for their applications.

## 1. Introduction

The microneedles (MNs) were first described as a novel method of drug delivery in 1998 [1]. In clinical scenarios, parenteral drug delivery is typically limited to intravenous (IV) injection, intramuscular (IM) injection, subcutaneous administration, or transdermal (topical) application. Of these, the IV method allows the greatest bioavailability and dosing control, but has the disadvantages of pain, infection, and complexity in maintaining venous access. IM alternative is often used for quick access and bolus delivery of analgesics, antipsychotics and peptide vaccines, but it has similar drawbacks. For drugs such as insulin or various immunotherapy agents, the subcutaneous method is preferred, but pain, variable pharmokinetics and limited bioavailability are considerable issues. Other routes of administration, such as nasal or inhalational delivery, often raise concerns of dosage reproducibility and local effects. Finally, topical routes are limited because a relatively small number of agents are available, and many of these are used in the dermatological field. This route is limited primarily by the ability of pharmacologic agent to diffuse through the outer-most layer of skin, the stratum corneum [2]. Physical and chemical properties known to influence the abilities of molecules to penetrate the skin include liposolubility/hydrophilicity, molecular weight, ionization, carrier nature, and dilution ratio [3].

Microneedles are typically described as being long enough to penetrate the epidermis (and the outermost stratum corneum), but not to a depth that stimulates the nociceptive nerve endings within the dermis [4]. They also possess shorter, narrower geometries that facilitate pain prevention [5]. Additionally, MNs potentially direct the administration of agents to the dermis, a layer that possesses rich vascular and lymphatic perfusion. This layer also possesses unique pharmacokinetic and pharmacodynamic properties, and these are currently being investigated for systemic purposes [6].

In general, several types of MNs systems are used for drug delivery. Solid MNs can first pierce the skin, creating pores. After removal, a gel formulation or transdermal patch is placed over the area to facilitate delivery. Alternatively, MNs can be first coated with the agent and then applied to the skin [7]. Biodegradable MNs that encapsulate drugs are usually meant to detach within the skin, facilitating controlled drug release [8]. Hollow MNs are used to deliver drugs using a concept similar to injection, but using physical forces that include both diffusion and pressure [9]. Microneedles have been fabricated from an assortment of materials, such as silicon, metal, biodegradable or non-biodegradable polymers, and glass [10]. Their small size presents drawbacks, such as a limited total delivered dose and an extended time necessary for that dose to be transported into the dermis [8]. Drug formulations must be carefully modified for this purpose.

Our previous studies had suggested that two-step fabrication of MN patches had several benefits [11,12]. To evaluate the ability of a patch comprised of two-layer dissolving MN patches composed of gelatin and sodium carboxymethyl cellulose (CMC) for drug delivery, we developed and utilized diabetic mice and human cadaveric skin models [13]. An increasing number of articles have proposed new methods of MN patches fabrication and utilized animal models to evaluate their effects, but relatively few papers have discussed the effects on human cadaveric skin. Since the final goal of MNs is for clinical applications, a key highlight of this study was to provide preliminary information on MNs use on human skin, apply conventional techniques to evaluate the effect of MNs, and propose an ideal anatomic location of human skin for future clinical application of MN patches.

## 2. Materials and Methods

### 2.1. Materials

Commercially available MN patches were purchased from 3M^TM^ (3M, St. Paul, MN, USA), and negative molds were manufactured from polydimethulsiloxane (PDMS; Sylgard 184, Dow Corning, Belgium). Gelatin (porcine skin, type A, 90–110 Bloom), CMC (MW 90 kDa), rhodamine 6G (R6G; MW 470.01 Da), fluorescein 5(6)-isothiocyanate (FITC; MW 389.38 Da), and insulin (from bovine pancreas, 25 U/mg, MW approximately 5.73 kDa) were purchased from Sigma-Aldrich (St. Louis, MO, USA).

### 2.2. Preparation of Dissolving Gelatin/CMC MN Patches

The negative moldings used to fabricate MN patches were prepared from master templates of 3M^TM^ MN patches, as described elsewhere [12]. In brief, a modified two-step process was used [14,15] where a 10% gelatin solution was loaded with a drug and poured into each PDMS mold, then centrifuged at 4000 rpm for 30 min to fill the mold cavities. Residual solutions on the mold surface was removed, then a drug-free 10% CMC solution was placed on the top of 10% gelatin and centrifuged at 4000 rpm for 10 min. All two-layered gelatin/CMC MN patch molds were then dried at room temperature overnight.

### 2.3. Fabrication of Drug-Loaded MN Patches

A water-soluble red fluorescent dye, R6G, was used as a low-molecular-weight model drug in this study. The dye was dissolved in deionized water to prepare a 0.5 mg/mL stock solution. Each polymer solution was augmented with 50 mL stock solution. Insulin-FITC dissolved in 0.1 M HCl was added to the gelatin solutions and mixed in uniform proportions to obtain insulin-containing gelatin solutions. An inverted fluorescence microscopy was used to observe the shapes of the drug-loaded MNs and to confirm that the drugs were successfully loaded into the needle tips of the MN patches.

### 2.4. Ex Vivo Penetration Tests in Mouse Skin

To evaluate the insertion capabilities of the proposed gelatin/CMC MNs, patches of MN were inserted to mouse cadaveric skins using a handmade applicator and 9 N of force (similar to that applied by the thumb) for 10 min. They were then removed, and the morphological changes of the MNs in the patches were observed over time using scanning electron microscopy (SEM; S-3000N, Hitachi, Tokyo, Japan).

### 2.5. Optical Coherence Tomography and Imaging of Transdermal Delivery

Optical coherence tomography (OCT; Chang Gung University, Taiwan) was used to assess the depth of MN penetration into the mouse skins in real-time before the patches were removed (during the 10-min application time). Patches loaded with R6G were also applied to mouse skins to visualize the transdermal delivery under 9 N of force over 60 min. The depth of the skin, from the surface to the dermis in the vertical direction, was determined using confocal microscopy.

### 2.6. In Vivo Transdermal Delivery via Insulin-Loaded MNs

The in vivo imaging system (IVIS; Xenogen 200, Caliper Life Sciences, Alameda, CA, USA) was used as a non-invasive analytical tool to visualize cutaneous permeation of insulin in live animals. Diabetic mice under anesthesia were imaged at 10 min, 1 h, and 3 h after treatment with MN patches loaded with or without (control) FITC–insulin. The IVIS in this study was used to analyze all fluorescence data, which were presented as photon flux (photons sec^−1^cm^−2^ steradian^−1^) [16].

### 2.7. Transdermal Insulin Delivery via MN Patches

The protocols used in the animal studies were approved by the Institutional Animal Care and Use Committee of Chang Gung Memorial Hospital, and we conducted the experiments based on the guidelines of the Laboratory Animal Center of Chang Gung Memorial Hospital. Eight-week-old male *db/db* mice (C57BL/6) were used for this study. The average blood sugar level of each mouse exceeded 600 mg/dL at study inception. All diabetic mice were randomly divided into three groups (n = 6 for each group): the control group, which received unloaded MNs applied to their backs and stabilized with tape; the SC group, which received insulin injections (0.2 IU) subcutaneously into the abdominal skin using a hypodermic needle; and the insulin-loaded MN group, which received insulin via insulin-loaded MNs (0.2 IU per patch) applied to their backs and stabilized with tape. Blood samples were collected 0, 1, 2, 3, 4, 5, and 6 h after treatment. The percent change in plasma glucose levels at each time point in time was calculated based on the initial value.

### 2.8. Tissue-Marking Dye Staining and OCT Measurement of Human Cadaveric Skin

Human cadaveric skin was obtained from different anatomic locations from patients. All patients signed an informed consent. This study is specifically approved by the Institutional Review Board of the Chang Gung Medical Foundation, Taiwan (IRB Nos. 103-3234B and 104-0046C). Subcutaneous fat was removed after harvesting the skin, then the full-thickness skin was stored at −20 °C until use. Various samples (dorsal ear skin, volar forearm skin, medial thigh skin and lower abdomen skin) were obtained from donors and used to demonstrate reproducibility. Each skin was thawed to room temperature and dabbed with clean tissue paper to remove excess moisture before use.

To evaluate the skin insertion capabilities of the gelatin/CMC MN patches into human cadaveric skin, a homemade applicator was used, applying 9 N of force for 10 min. Upon patch removal, the skin surface was exposed to a blue tissue-marking dye for 1 min, revealing the insertion sites [9]. The blue spots were then observed using a stereomicroscope (P6000, Nikon, Tokyo, Japan) and photographed. OCT measurement was also used for real-time assessment of penetration depth in human cadaveric skins of the gelatin/CMC MN patches with an application force of 9 N for 10 min without peeling off.

## 3. Results and Discussion

### 3.1. Preparation and Characterization of Gelatin/CMC MN Patches

**Figure 1:**
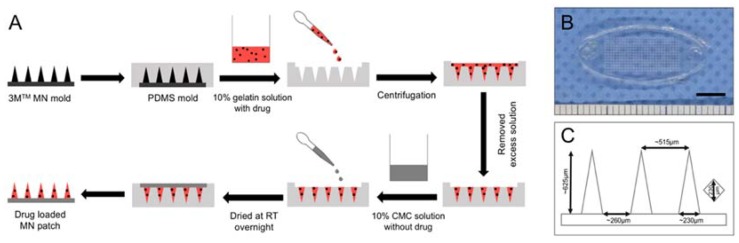
(**A**) Schematics of the fabrication process of dissolving gelatin/CMC MN patches. The drug was localized in the tip of the needles. (**B**) Gross view of the gelatin/CMC MN patch. Scale bar = 5 mm. (**C**) Average dimensions of geometrical parameters of gelatin/CMC MN patches.

As described previously, the two-step casting process was used to localize the drugs at the needle tip and to minimize drug waste after insertion [11,12]. The two-step casting procedure was superior than the one-step because we may choose materials (e.g., gelatin) that provide the hardness of needle tips for easier penetration into the skin and other materials (e.g., CMC) that are more flexible for the backing layer of MNs [17,18]. The two-step preparations were also beneficial to localize the protein drug in the needle tips to minimize the drug waste during the fabrication process [11,19,20]. Figure 1A demonstrated the fabrication process of dissolving gelatin/CMC MN patches that we used for this study. Our MN patches were replicated from the 3M molds. Figure 1B,C showed the detailed dimensions (measured by ImageJ software) and the geometric parameters of the gelatin/CMC MN patches.

### 3.2. Insertion Capability of Gelatin/CMC MN Patches in Mice Skin

**Figure 2:**
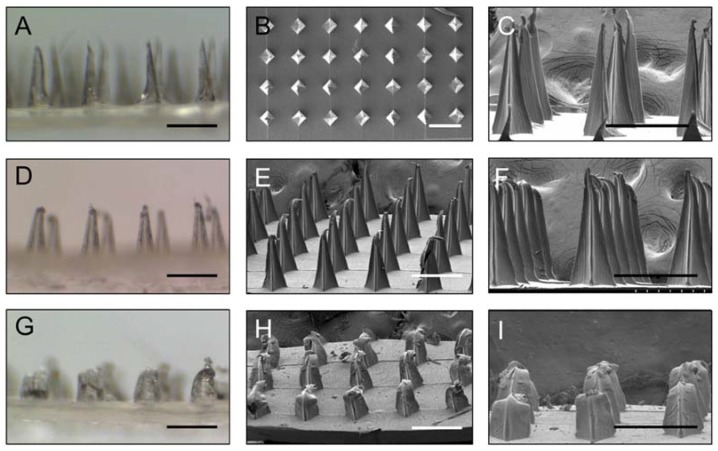
(**A**) Stereomicroscopy images and (**B**,**C**) scanning electron microscopy images of gelatin/CMC MNs before insertion. (**D**–**F**) The MNs 10 min after insertion. (**G**–**I**) The MNs 30 min after insertion. Scale bar = 500 μm.

Gelatin/CMC MN patches were punched into mice cadaveric skin with an application force of 9 N to test the skin insertion capabilities. Under SEM, the morphological changes of the MNs after insertion for 0, 10, 30 and 60 min were investigated (Figure 2). The findings show that the needles dissolved over time: more than 50% at 30 min and almost completely after 60 min (data not shown). The collective results from the mice skin penetration test and the morphological changes show that the gelatin/CMC MNs can successfully penetrate the skin barrier and release their contents after dissolving.

### 3.3. Optical Coherence Tomography and Confocal Microscopic Demonstrations of Penetration Depth and Position

Optical coherence tomography (OCT) is a non-invasive and real-time imaging technique that can be used to evaluate the skin in vivo before and after puncture test in live animals [21]. In this study, OCT was used to observe the exact depth of the MN patches penetration into mice cadaveric skin. Unlike stereomicroscopy and SEM, OCT can not only confirm the penetration of MNs into mouse skin, but it also provides information about the cross-section imaging and the true depth of penetration. Figure 2A,B shows real-time OCT images of intact mouse skin and gelatin/CMC MNs insertion into mouse skin. White arrows indicate the sites of micro-disruptions in the skin, displaying the ability of MNs to puncture the stratum cornea barrier. These results indicate that the gelatin/CMC MNs fabricated from the 3M mold possess the capability to penetrate the mice skin and the stratum cornea to create micro-holes, which is consistent with those reported elsewhere [12].

**Figure 3:**
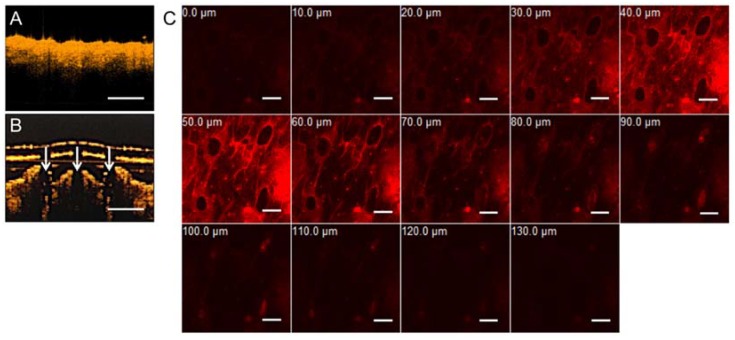
In vitro transdermal delivery of R6G in mice via patches of gelatin/CMC MNs. (**A**) OCT image of mice skin before MNs insertion. (**B**) OCT image of mice skin after insertion. White arrows indicate the location of micro-disruption in the dermis. Scale bar = 500 μm. (**C**) The R6G-loaded gelatin/CMC MNs penetrate mice skin at certain depths after insertion for 60 min. Scale bar = 200 μm.

In order to visualize the details during transdermal delivery via MNs, patches of R6G-loaded gelatin/CMC MNs were applied to a mouse cadaveric skin using 9 N of force, then removed after 60 min. The vertical depths of penetration from the skin surface were determined using confocal microscopy. As shown in Figure 3C, the maximum drug diffusion depths of R6G was approximately 130 μm. The in vitro transdermal delivery results indicate that the MNs can be used to facilitate penetration of hydrophilic and high-molecular-weight proteins in penetrating the stratum cornea, enhancing drug diffusion in the skin.

### 3.4. In Vivo Study of Transdermal Delivery of FITC-Insulin from MNs

**Figure 4:**
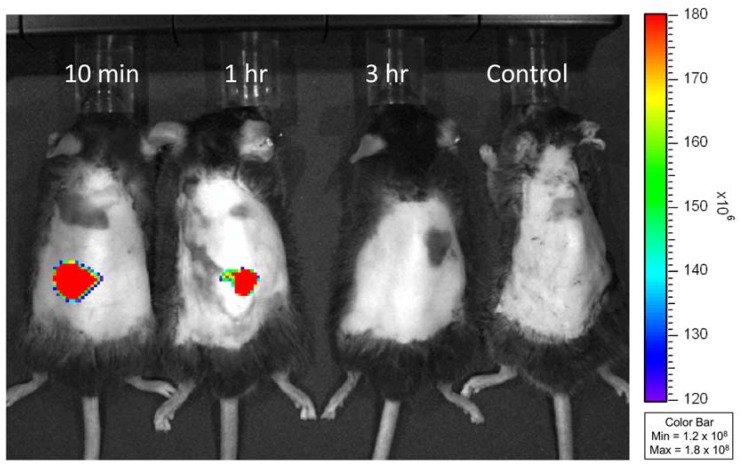
In vivo fluorescence images of *db/db* mice at 10 min, 1 h and 3 h after treatment of FITC-insulin-loaded gelatin/CMC MN patches and unloaded (control) MN patches. The insertion sites of mice dorsal skin presented a strong fluorescent signal at 10 min indicating that the dissolving MNs rapidly released encapsulated FITC-insulin. The fluorescence intensity decreased at 1 h and disappeared at 3 h after application of MN patches.

In order to characterize the skin permeation of FITC-insulin from loaded MNs, patches with and without insulin were applied to mice for 10 min before removal. Using the IVIS, images were captured 10 min, 1 h and 3 h after treatment. As shown in Figure 4, mice receiving the FITC-insulin-loaded MNs gave off a strong fluorescent signal 10 min after treatment, which decreased 1 h after treatment of MN patches. These results indicate that the gelatin/CMC MNs fast released encapsulated FITC-insulin which gradually diffused into the mice skin. This finding is similar to the previous report [22], which showed that the fluorescence intensity of FITC-insulin noticeably decreased 1 h after application of the loaded MNs.

### 3.5. Transdermal Delivery of Insulin to Diabetic Mice

**Figure 5:**
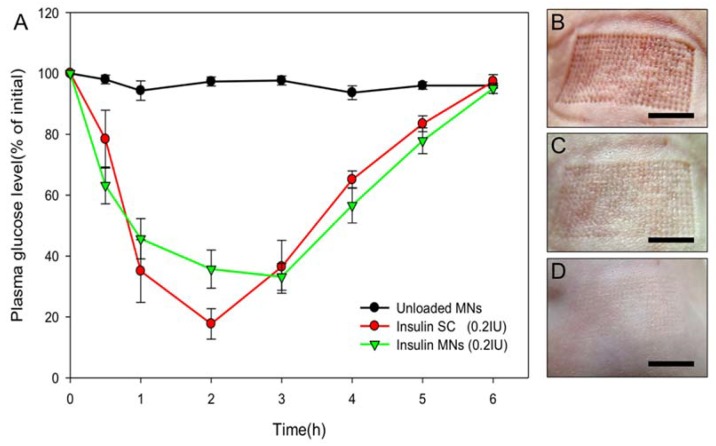
(**A**) Plasma glucose level changes over time in *db*/*db* mice after receiving an unloaded or insulin-loaded MN patches or a subcutaneous injection of insulin. (n = 6 per group). Skin puncture marks after (**B**) 10 min, (**C**) 1 h, and (**D**) 3 h. Scale bar = 5 mm.

The gelatin/CMC MNs patches were used in *db/db* mice to compare the effects of insulin delivered transdermally to insulin delivered subcutaneously [23]. Figure 5 shows the plasma glucose levels of the two treatment groups and the control. A rapid decrease is seen in the plasma glucose levels after treatment in the SC group. The maximal decrease was when the plasma glucose level was approximately 80% of its initial value. Blood sugar levels returned to their initial values 6 h after treatment. A similar blood sugar change was observed in the MN group after treatment with the same dose of insulin, but the maximum decrease in plasma glucose level occurred when it was approximately 70% of its initial value. The MN group reached its lowest plasma glucose levels slightly later than the SC group [24,25]. No hypoglycemic effects were detected in the controls.

The maximum glucose level change appeared 1 h after treatment of application in both the SC group and the MN group. In the former, the blood sugar continued to decrease, reaching its lowest level after 2 h, but in the MN group, the minimum level was reached after 3 h. These results suggest that the MN patch may produce a more stable insulin level in plasma. The gelatin/CMC MN patches provide an easy, quick, and cost-effective way for insulin delivery, improving bioavailability compared to that found in other studies [11]. The in vivo results prove that the fabrication of the gelatin/CMC MN patches allow gradual and moderate decreases in blood sugar and that physiologic temperature is suitable for the current drug encapsulation and maintenance of the protein activity. Since the MN patches were designed based on insulin dosages suitable for clinical use, a proposed concept for human use would be increasing the number of patches according to patient blood sugar level for improved blood sugar control.

### 3.6. Human Cadaveric Skin Penetration Test

**Figure 6:**
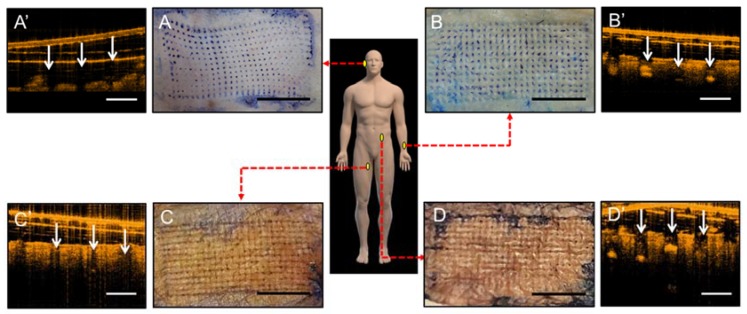
In vitro insertion capability of patches of gelatin/CMC MNs applied to discarded human skins from various locations which were then stained with blue tissue-marking dye after patches removal. (**A**) Dorsal ear skin. (**B**) Ventral forearm skin. (**C**) Medial thigh skin. (**D**) Abdominal wall skin. Scale bar = 5 mm. (**A**’–**D**’) Respective OCT images of human cadaveric skin after MNs insertion. White arrows indicate the location of micro-disruption in the dermis. Scale bar = 500 μm.

Since MN patches were designed to deliver drugs into the skin for clinical purposes, human cadaveric skin was used to determine the ideal anatomical location for their application. Human skin can be retrieved from donors as waste tissues from plastic and reconstructive surgeries. Although preserved through hypothermia, they serve as good representatives of human skin in the living individual, especially in their aspects of micro- and macro-anatomy [26,27,28]. Parameters, such as temperature, preservation method, age of donor, and harvest location, affect the ability of skin to model living tissue. Human cadaveric skin has been shown to be excellent models for barrier integrity and water permeability and have been used to test transdermal and controlled drug release through the use of MNs [10]. In this study, various samples of human cadaveric skin were obtained [29], and a tissue-staining dye revealed the depth of penetration of the MNs into the dermal layer, as shown in Figure 6. Penetration rate at the posterior auricular and forearm skin were approximately 100%. Penetration rate at the medial thigh skin reached ~87% and lower abdominal skin penetration rate was ~78%. OCT image comparison between human skin and mice skin demonstrated that dorsal ear and volar forearm skin thickness and penetration depth were closer to mice skin, indicating that the clinical effects of insulin-loaded patches could potentially be obtained in humans if applied to these locations [30]. Although dorsal ear had the thinnest skin thickness compared to the other samples, volar forearm skin seems to be the more ideal anatomical location for MN patches in future clinical application due to its ease of application, flat topography for uniform penetration and relatively thin skin thickness [31,32]. We used the same insulin drug on the gelatin/CMC MN patches for animal study as we would on clinical patients. Clinically, the dosage of insulin given to each patient is dependent on each individual’s sugar levels. Therefore, we can easily increase the number of our MN patches used to achieve sugar control of patients in clinical applications [30].

## 4. Conclusions

Microneedle patches can be successfully used in diabetic mice and a human cadaveric skin models. A gelatin/CMC MN patch fabricated via a simple two-layer process is an ideal alternative to conventional insulin injection. It provides an easy, convenient approach and is an efficient method for controlling blood sugar in *db/db* diabetic mice. Insulin retains its pharmacological activity after release from MNs, producing a significant hypoglycemic effect in diabetic mice. Human cadaveric skin tests show that the skin at the volar aspect of the forearm is potentially the ideal location for the clinical application of MN patches. These collective results suggest that dissolving gelatin/CMC MNs are potentially promising devices for the transdermal delivering various biomolecules.

## References

[1] Henry S., McAllister D.V., Allen M.G., Prausnitz M.R. (1998). Microfabricated microneedles: A novel approach to transdermal drug delivery. J. Pharm. Sci..

[2] Paudel K.S., Milewski M., Swadley C.L., Brogden N.K., Ghosh P., Stinchcomb A.L. (2010). Challenges and opportunities in dermal/transdermal delivery. Ther. Deliv..

[3] Ita K.B. (2015). Chemical penetration enhancers for transdermal drug delivery—Success and challenges. Curr. Drug Deliv..

[4] Xie X., Pascual C., Lieu C., Oh S., Wang J., Zou B., Xie J., Li Z., Xie J., Yeomans D.C. (2017). Analgesic microneedle patch for neuropathic pain therapy. ACS Nano.

[5] Kim Y.C., Park J.H., Prausnitz M.R. (2012). Microneedles for drug and vaccine delivery. Adv. Drug Deliv. Rev..

[6] Pettis R.J., Harvey A.J. (2012). Microneedle delivery: Clinical studies and emerging medical applications. Ther. Deliv..

[7] Milewski M., Brogden N.K., Stinchcomb A.L. (2010). Current aspects of formulation efforts and pore lifetime related to microneedle treatment of skin. Expert Opin. Drug Deliv..

[8] Park J.H., Allen M.G., Prausnitz M.R. (2006). Polymer microneedles for controlled-release drug delivery. Pharm. Res..

[9] Burton S.A., Ng C.Y., Simmers R., Moeckly C., Brandwein D., Gilbert T., Johnson N., Brown K., Alston T., Prochnow G. (2011). Rapid intradermal delivery of liquid formulations using a hollow microstructured array. Pharm. Res..

[10] McAllister D.V., Wang P.M., Davis S.P., Park J.H., Canatella P.J., Allen M.G., Prausnitz M.R. (2003). Microfabricated needles for transdermal delivery of macromolecules and nanoparticles: Fabrication methods and transport studies. Proc. Natl. Acad. Sci. USA.

[11] Lee I.C., Lin W.M., Shu J.C., Tsai S.W., Chen C.H., Tsai M.T. (2017). Formulation of two-layer dissolving polymeric microneedle patches for insulin transdermal delivery in diabetic mice. J. Biomed. Mater. Res. Part A.

[12] Lee I.C., He J.S., Tsai M.T., Lin K.C. (2015). Fabrication of a novel partially dissolving polymer microneedle patch for transdermal drug delivery. J. Mater. Chem. B.

[13] Gala R.P., Zaman R.U., D’Souza M.J., Zughaier S.M. (2018). Novel Whole-Cell Inactivated Neisseria Gonorrhoeae Microparticle Vaccine Formulation in Microneedles for Transdermal Immunization. https://europepmc.org/abstract/ppr/ppr49728.

[14] McGrath M.G., Vucen S., Vrdoljak A., Kelly A., O’Mahony C., Crean A.M., Moore A. (2014). Production of dissolvable microneedles using an atomised spray process: Effect of microneedle composition on skin penetration. Eur. J. Pharm. Biopharm..

[15] Guo L., Chen J., Qiu Y., Zhang S., Xu B., Gao Y. (2013). Enhanced transcutaneous immunization via dissolving microneedle array loaded with liposome encapsulated antigen and adjuvant. Int. J. Pharm..

[16] Lee K., Kim J.D., Lee C.Y., Her S., Jung H. (2011). A high-capacity, hybrid electro-microneedle for in-situ cutaneous gene transfer. Biomaterials.

[17] Fukushima K., Ise A., Morita H., Hasegawa R., Ito Y., Sugioka N., Takada K. (2011). Two-layered dissolving microneedles for percutaneous delivery of peptide/protein drugs in rats. Pharm. Res..

[18] Ito Y., Hirono M., Fukushima K., Sugioka N., Takada K. (2012). Two-layered dissolving microneedles formulated with intermediate-acting insulin. Int. J. Pharm..

[19] González-Vázquez P., Larrañeta E., McCrudden M.T., Jarrahian C., Rein-Weston A., Quintanar-Solares M., Zehrung D., McCarthy H., Courtenay A.J., Donnelly R.F. (2017). Transdermal delivery of gentamicin using dissolving microneedle arrays for potential treatment of neonatal sepsis. J. Control. Release.

[20] Liu S., Jin M.-N., Quan Y.-S., Kamiyama F., Katsumi H., Sakane T., Yamamoto A. (2012). The development and characteristics of novel microneedle arrays fabricated from hyaluronic acid, and their application in the transdermal delivery of insulin. J. Control. Release.

[21] Coulman S.A., Birchall J.C., Alex A., Pearton M., Hofer B., O’Mahony C., Drexler W., Povazay B. (2011). In vivo, in situ imaging of microneedle insertion into the skin of human volunteers using optical coherence tomography. Pharm. Res..

[22] Ling M.-H., Chen M.-C. (2013). Dissolving polymer microneedle patches for rapid and efficient transdermal delivery of insulin to diabetic rats. Acta Biomater..

[23] Kitada M., Ogura Y., Koya D. (2016). Rodent models of diabetic nephropathy: Their utility and limitations. Int. J. Nephrol. Renovasc. Dis..

[24] Kalluri H., Banga A.K. (2011). Formation and closure of microchannels in skin following microporation. Pharm. Res..

[25] Siddiqui O., Sun Y., Liu J.C., Chien Y.W. (1987). Facilitated transdermal transport of insulin. J. Pharm. Sci..

[26] Wermeling D.P., Banks S.L., Huclson D.A., Gill H.S., Glupta J., Prausnitz M.R., Stinchcom A.L. (2008). Microneedles permit transdermal delivery of a skin-impermeant medication to humans. Proc. Natl. Acad. Sci. USA.

[27] Gill H.S., Prausnitz M.R. (2007). Coated microneedles for transdermal delivery. J. Control. Release.

[28] Park J.H., Allen M.G., Prausnitz M.R. (2005). Biodegradable polymer microneedles: Fabrication, mechanics and transdermal drug delivery. J. Control. Release.

[29] Chan J.C., Ward J., Quondamatteo F., Dockery P., Kelly J.L. (2014). Skin thickness of the anterior, anteromedial, and anterolateral thigh: A cadaveric study for split-skin graft donor sites. Arch. Plast. Surg..

[30] Ripolin A., Quinn J., Larrañeta E., Vicente-Perez E.M., Barry J., Donnelly R.F. (2017). Successful application of large microneedle patches by human volunteers. Int. J. Pharm..

[31] Ha R.Y., Nojima K., Adams W.P., Brown S.A. (2005). Analysis of facial skin thickness: Defining the relative thickness index. Plast. Reconstr. Surg..

[32] Shuster S., Black M.M., McVitie E. (1975). The influence of age and sex on skin thickness, skin collagen and density. Br. J. Dermatol..

